# Marinesco–Sjögren Syndrome: A Novel *SIL1* Variant with In Silico Analysis and Review of the Literature

**DOI:** 10.3390/life15121855

**Published:** 2025-12-02

**Authors:** Elif Sibel Aslan, Sajjad Eslamkhah, Nermin Akcali, Cuneyd Yavas, Lutfiye Karcioglu Batur, Esma Sengenc, Adnan Yuksel

**Affiliations:** 1 Department of Molecular Biology and Genetics, Faculty of Engineering and Natural Sciences, Biruni University, Istanbul 34460, Türkiye; nakcali@biruni.edu.tr (N.A.); cyavas@biruni.edu.tr (C.Y.); lbatur@biruni.edu.tr (L.K.B.); 2 Department of Child Neurology, Faculty of Medicine, Cerrahpaşa University, Istanbul 34098, Türkiye; dresmasengenc@gmail.com; 3 Department of Pediatrics, Faculty of Medicine, Biruni University, Istanbul 34010, Türkiye; ayuksel@biruni.edu.tr

**Keywords:** Marinesco–Sjögren syndrome, *SIL1*, splice-site variant, whole-exome sequencing, genotype–phenotype correlation, structural modeling, rare neurogenetic disorder

## Abstract

Marinesco–Sjögren syndrome (MSS) is a rare autosomal recessive disorder characterized by cerebellar ataxia, congenital cataracts, developmental delay, hypotonia, and progressive myopathy. Most reported cases are linked to pathogenic variants in *SIL1*, a gene encoding a co-chaperone essential for protein folding in the endoplasmic reticulum. Here, we present a comprehensive case study of a Turkish pediatric patient diagnosed with MSS, supported by genetic, bioinformatic, and structural modeling analyses. Whole-exome sequencing revealed a homozygous splice-site variant (*SIL1* c.453+1G>T), confirmed by Sanger sequencing and segregation analysis. In silico annotation using Genomize, InterVar, Franklin, VarSome, ClinVar, OMIM, and PubMed classified the variant as pathogenic according to ACMG guidelines. Structural modeling by Phyre2 and I-TASSER demonstrated that the variant abolishes the intron 5 donor site, leading to truncation of the wild-type 461-amino-acid protein into a shortened ~189-amino-acid polypeptide. This truncation results in the loss of critical Armadillo (ARM) repeats required for HSPA5 interaction, explaining the observed instability and impaired chaperone function. Clinically, the patient presented with congenital cataracts, ataxia, developmental delay, and progressive muscle weakness, consistent with previously reported MSS cases. Comparison with the literature confirmed that splice-site variants frequently correlate with severe phenotypes, including early-onset ataxia and cataracts. This report highlights the importance of integrating genomic, structural, and clinical data to better understand genotype–phenotype correlations in MSS. Our findings expand the mutational spectrum of *SIL1*, reinforce the role of splicing defects in disease pathogenesis, and emphasize the necessity of comprehensive molecular diagnostics for rare neurogenetic syndromes.

## 1. Introduction

Marinesco–Sjögren syndrome (MSS) is a rare genetic disorder characterized by a range of clinical features affecting multiple tissues. Common manifestations include congenital or early childhood cataracts, muscle weakness (myopathy), and ataxia, which can impair mobility and lead to progressive muscle function decline [1,2]. Intellectual disability, typically mild to moderate, and skeletal abnormalities such as short stature and scoliosis are also frequently observed [1].

Additional symptoms may include strabismus, nystagmus, and dysarthria. Hypergonadotropic hypogonadism, resulting in delayed or absent puberty, is another common finding. MSS is primarily caused by variants in the *SIL1* gene, which encodes a protein essential for proper protein folding within the endoplasmic reticulum (ER) [3]. These variants lead to a dysfunctional SIL1 protein, disrupting protein folding and causing the accumulation of misfolded proteins, which damages various tissues. However, around one-third of individuals with MSS do not have detectable *SIL1* variants, leaving their etiology unclear. MSS is inherited in an autosomal recessive manner, with over 100 cases documented worldwide, and affected individuals’ parents are typically asymptomatic carriers of the mutated gene [4].

SIL1 is a 461-amino acid protein featuring a potential signal sequence at its NH2-terminus and a tetrapeptide at its COOH-terminus that likely serves as an ER retrieval sequence. This protein is widely expressed and functions as an adenine nucleotide exchange factor for GRP78, a key heat-shock protein chaperone in the ER. SIL1 regulates GRP78’s ATPase cycle, influencing protein translocation into the ER, proper folding of newly synthesized proteins, and degradation of misfolded proteins [5,6]. The 17 MSS-associated variants in *SIL1*, including nonsense, frameshift, or splice site alterations, lead to loss of protein function through premature termination or abnormal splicing, with one reported missense variant. MSS in some patients without detectable *SIL1* variants indicates genetic variability [7]. While most *SIL1* pathogenic variant-positive MSS patients exhibit hallmark clinical features such as intellectual disability (ID), myopathy, cerebellar atrophy, ataxia, and cataracts, the additional features and their severity can vary between patients, seemingly depending on the type of variant. Approximately 40% of patients with classical MSS do not have identifiable *SIL1* gene variants, highlighting the clinical and genetic heterogeneity of the disorder [4,8]. The disease’s pathogenesis is linked to defective protein quality control in the ER, where SIL1 dysfunction impairs GRP78 activity, causing disrupted protein folding and accumulation of unfolded proteins. This accumulation results in ER stress, which can lead to cell death. MSS is thus categorized as a protein misfolding and accumulation disorder due to defective chaperone function. In MSS, severe cerebellar atrophy is observed, primarily due to the loss of Purkinje and granule cells in all cerebellar lobules except the vestibulocerebellum, with remaining Purkinje cells often vacuolated or binucleated. This hypothesis is supported by studies on the SIL1-KO (*SIL1* gene knockout), which exhibits SIL1 loss-of-function, leading to an unfolded protein response, abnormal protein accumulation, and neurodegeneration in cerebellar Purkinje cells, culminating in ataxia [9].

In this study, we aim to comprehensively investigate the phenotypic characteristics and genetic findings of a patient diagnosed with MSS. Using in silico bioinformatic tools, we will evaluate the pathogenicity and structural impact of the identified genetic variant to better understand its potential functional consequences. Furthermore, we aim to correlate the molecular findings with the patient’s clinical presentation, providing a genotype–phenotype interpretation. In addition, a comparative analysis with previously reported MSS cases in the literature will be conducted to assess the spectrum of variant types, molecular mechanisms, and associated clinical outcomes, thereby contributing to the understanding of the genetic and phenotypic diversity of MSS.

## 2. Materials and Methods

### 2.1. Ethical Approval

In this study, a consent form was obtained from the patient/patient’s legal guardian. This research is conducted at Biruni University with ethical approval number 2024-BIAEK/04-26 granted by the ethics committee of Biruni university.

### 2.2. Bioinformatic Modeling and Analysis of Wild Type and Mutant of SIL1

*SIL1* (HGNC ID:24624) sequence information of Homo sapiens was obtained from the NCBI database (NM_022464.5). *SIL1* c.453+1G>T variant data were generated with the Phyre2 and I-TASSER bioinformatics tools [10,11]. Proteins identified by the prediction algorithms were predicted again in ModRefiner to see improvements in both global and local structures, with more accurate side chain positions, better hydrogen bond networks and less atomic overlap [12]. In this study, a scenario-based bioinformatic assessment was conducted to evaluate the potential impact of the c.453+1G>T variant identified in the *SIL1* gene. SpliceAI was applied to predict splice-site alterations and to determine the likelihood of donor-site disruption. The algorithm indicated a high probability of canonical donor loss, generating two distinct theoretical outcomes. Superimpose and conformational analysis of wild type and mutant proteins were performed with PyMOL (ver2.5.5).

### 2.3. Genetic Analysis

Genomic DNA was isolated from peripheral blood, and whole-exome sequencing (WES) was performed using the Twist Human Comprehensive Exome kit to capture coding regions and splice sites. Following library enrichment and quality control, samples were sequenced as 100 bp paired-end reads at a mean depth of ~100× on the BGI platform (Shenzhen, China). Variants were annotated and interpreted using Genomize (v6.14.4), InterVar, Franklin, VarSome, ClinVar, OMIM, and PubMed. Variants with minor allele frequency (MAF) >0.1% in population databases (e.g., gnomAD) were excluded. Pathogenicity was assessed with standard prediction algorithms and classified as pathogenic, likely pathogenic (LP), or variant of uncertain significance (VUS) according to ACMG consensus criteria.

## 3. Results

### 3.1. Patient Presentation and Genetic Study

A 21-year-old male patient is being followed up with a diagnosis of MSS. There is consanguinity between the parents. The mother was 22 years old and a housewife at the time of delivery, while the father was 24 years old. The patient was delivered at term through normal spontaneous delivery with no neonatal intensive care unit history and no complications at birth. Prenatal follow-up revealed no notable features. There is no family history of neurological disease, and he has a healthy 12-year-old sister. The mother has a history of one curettage and one stillbirth.

The patient had congenital hypotonia. Motor developmental milestones were markedly delayed—head control was achieved at 1.5 years, sitting at 1.5–2 years, and walking with support at 14–15 years—while cognitive development remained within normal limits. Under pediatric neurology follow-up, cranial MRI analysis performed in 2004 when the patient was 20 months old showed diffuse cerebellar vermian atrophic volume loss and cortical-dominant degenerative T2 FLAIR hyperintensity changes at the infratentorial level, suggesting pathologies characterized by cerebello-olivary degeneration, infantile cerebellar atrophy type, and cerebellar degeneration not affecting the spinal cord and pons. Cervical MRI was normal. The 2006 MRI showed diffuse cerebellar hypoplasia and vermian hypoplasia; the 2008 MRI revealed prominent cerebellar folia widths.

The patient underwent surgery for strabismus and cataract, requiring glasses due to lens replacement. He also had a tendon lengthening operation. Physical examination showed a head circumference of 52.5 cm (microcephaly), height of 170 cm, weight of 63 kg, clear consciousness, good cooperation and orientation, and a generally good condition. Cranial nerve examination revealed limited outward gaze in both eyes, more pronounced on the right, with nystagmus observed during eye movements. Other cranial nerves were normal. There were no signs of meningeal irritation. Deep tendon reflexes were absent. Muscle strength was 4/5 in the upper extremities and 3/5 in the lower extremities. There was no muscle atrophy, hypertrophy, or deformity. Skin examination was normal. Dysmorphic findings included synophrys, a short fourth toe on the left foot, and mild protruding ears. The patient had kyphoscoliosis. Cerebellar system examination showed dysmetria and dysdiadochokinesia. Tandem walking was ataxic with apparatus support. Speech was nasal and mildly dysarthric. There were no involuntary movements. Sensory examination was normal. Pectus excavatum was present. Loss-of-function variants in *SIL1*, which encodes the Nucleotide exchange factor SIL1, are known to be pathogenic. The variant identified in this study has not been previously reported in the literature (novel variant). In in silico analyses (MetaRNN, BayesDel addAF, Mutation taster, etc.) indicated that the variation (c.453+1G>T) causes a splice donor variant (Table 1). As a result of splice variant, the donor sequence GT > TT change in the intron region caused a change in the amino acid sequence and early termination that caused truncated protein.

**Figure 1:**
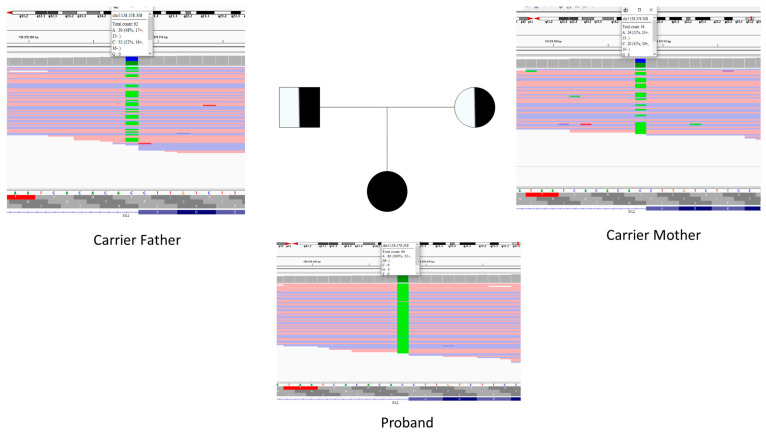
Pedigree chart of the family carrying *SIL1* variant. The pedigree demonstrates an autosomal recessive inheritance pattern. Both parents (I-1 and I-2) are healthy carriers (heterozygous, Aa). The proband (II-2), indicated by the arrow, is a male affected individual (homozygous recessive, aa). The sister (II-1) is an unaffected female with the wild-type genotype (AA).

The index variant’s computational evidence is summarized in Table 1. Segregation analysis was performed by NGS. The pedigree is presented in Figure 1.

### 3.2. Bioinformatic Results

SpliceAI has demonstrated that the mutation is highly likely to cause canonical donor loss, and that two alternative scenarios may arise in relation to this. Accordingly, it is assumed that either (A) a cryptic donor site within the exon is utilized, or (B) intron retention or exon skipping occurs due to the complete disruption of splicing. As no experimental validation was available within the scope of this study, a methodological approach based on hypothetical modeling was adopted to explain the RNA-level effect, and biological consequences were discussed, particularly with regard to scenario B. Although the cryptic donor usage scenario appears plausible according to SpliceAI outputs, the hypothetical model in this study is based on a mechanism where donor loss completely disrupts splicing, resulting in the complete or partial retention of intron 5 or the skipping of exon 5. In both cases, the reading frame is disrupted and the transcript encounters a premature stop codon (PTC) shortly thereafter. The resulting mRNA is likely degraded via the nonsense-mediated decay (NMD) pathway or leads to partial, truncated protein synthesis via escape transcription products. It is assumed that this truncated protein will lose its biological activity because it does not contain the functional domains of SIL1. Thus, it has been concluded that the c.453+1G>T change may be associated with severe loss of function.

**Figure 2:**
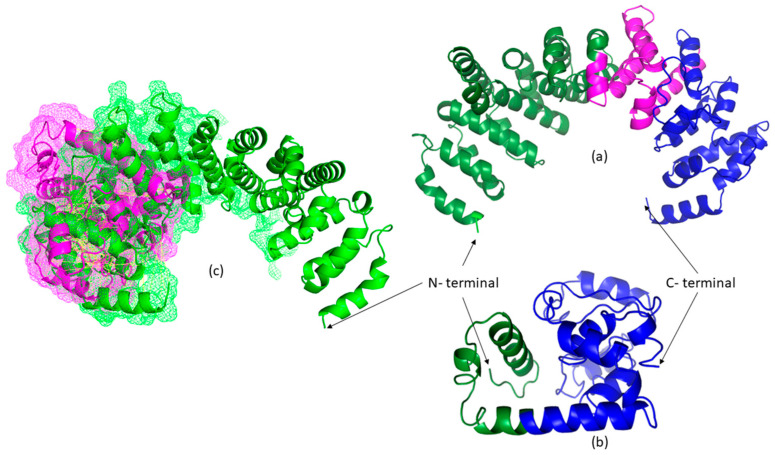
Illustration of the tertiary structure of SIL1. (**a**) Wild-type SIL1 protein prediction, the pink and green part indicates Armadillo-like helical, Nucleotide exchange factor Fes1 and Hsp70 Nucleotide Exchange Factors and Inhibitors domain regions. (**b**) Mutant-type SIL1 protein prediction. (**c**) Superimpose mesh representation of topological differences.

Currently, no experimentally resolved crystallographic or cryo-EM structure of the full-length human SIL1 protein is available. Consequently, structural interpretations of the c.453+1G>T variant rely on in silico predictions derived from homology-based modeling and domain-level analyses rather than empirically validated three-dimensional data. To assess structural consequences, SIL1 wild-type (WT) and SIL1 c.453+1G>T models were generated using Phyre2 and I-TASSER (Figure 2). The c.453+1G>T change is predicted to abolish the intron-5 donor site, causing aberrant splicing with a frameshift and premature truncation (~189 aa). This truncation removes most of the Armadillo (ARM) repeats (exons 6–10) that mediate HSPA5 engagement, providing a parsimonious rationale for reduced stability and loss of binding capacity in the truncated protein. We interpret C-scores as model-confidence metrics (not experimental quality labels) and treat these predictions as hypothesis-generating to be validated by RNA/functional studies.

**Figure 3:**
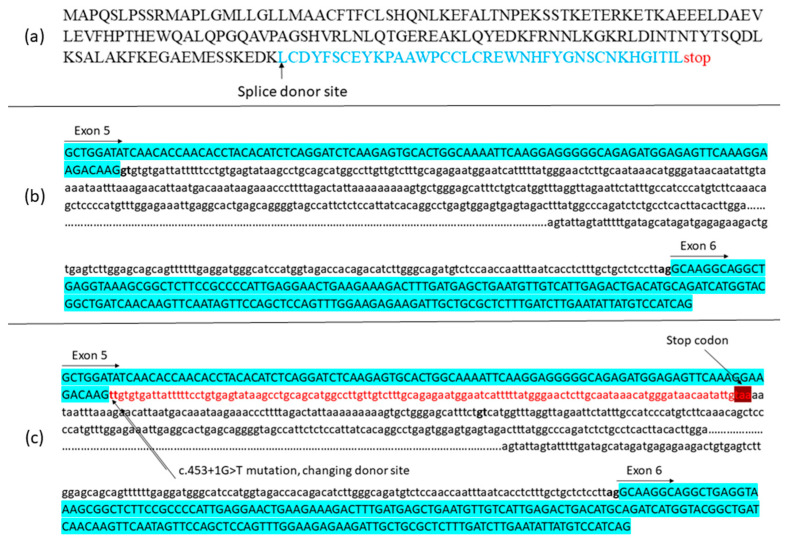
Predicted molecular consequences of the *SIL1* NM_022464.5:c.453+1G>T variant. (**a**) Amino acid sequence of the truncated SIL1 protein resulting from the premature termination. (**b**) Schematic representation of the canonical splicing site before the variant. Blue: exon 5–6 sequences; intronic region shown in lowercase. (**c**) Schematic representation of the aberrant splicing after the variant. Blue: exons 5–6; red: intronic sequence incorporated into the transcript leading to premature termination.

A positional change in splicing after the NM_022464.5: c.453+1G>T variant detected in the intron 5–6 splice donor site of *SIL1* is given in Figure 3. The variant changed the splice donor site in intron 5–6. The change in the splice region is suggested to cause an increase in the length of exon 5, while the stop codon following the additional 38 amino acids is expected to cause premature termination of the protein. The mature SIL1 protein, consisting of 461 amino acids, has been reduced to a small protein of 189 amino acids. The tertiary structure of SIL1 is unknown. In this study, we propose a model for the SIL1 tertiary structure for the first time The protein model of wild-type SIL1 had a C-score of −1.65. The wild type model was within the limits of x-ray quality. The C-score of the homology model of the 189 amino acid protein that emerged after the variant was −4.18. The mutant model was within the limits of NMR quality.

**Figure 4:**
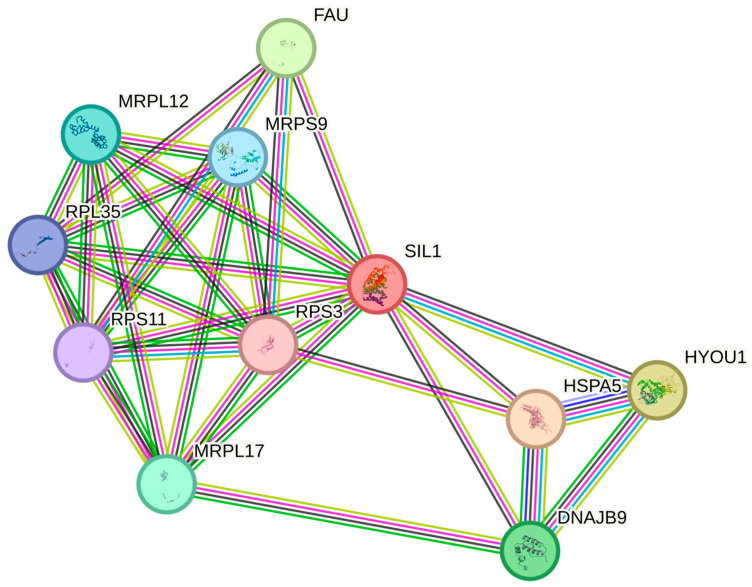
STRING-derived interaction network of SIL1 in Homo sapiens (confidence ≥0.70). Nodes represent proteins; edges represent evidence-weighted interactions (thicker lines indicate higher confidence). HSPA5 appears among the highest-confidence neighbors, consistent with SIL1’s role as a nucleotide-exchange factor for BiP within ER proteostasis [16].

The protein sequence of SIL1, which terminates early with variant, brought about the loss of protein structure including functional properties. The functional deficiency of *SIL1* will also disrupt the metabolic and regulatory processes with which it interacts. Elucidation of the molecular basis of MSS will be possible by a detailed analysis of the *SIL1* interaction map (Figure 4). The relationship between Endoplasmic reticulum chaperone BiP protein (HSPA5) in the interaction network created by considering the experimental data is remarkable.

**Figure 5:**
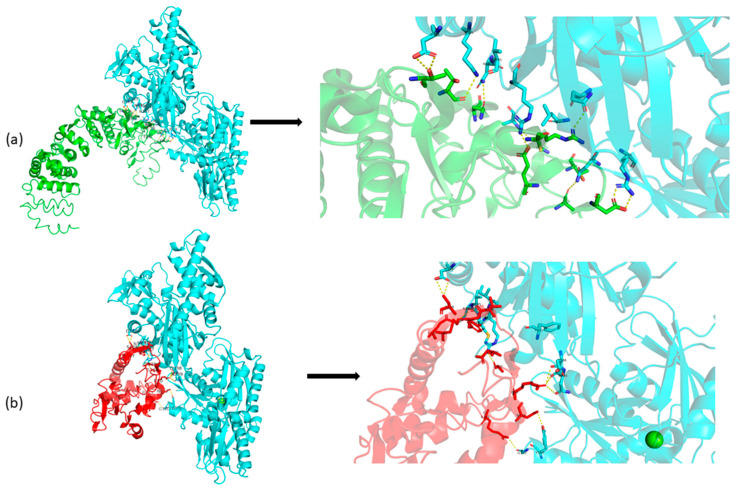
Significant changes were detected as a result of docking with HSP5A (cyan) gene protein, one of the chaperone proteins, before and after variant. (**a**). HSP5A gene protein docking model with wild type (green) (**b**). HSP5A gene protein docking model with mutant type (red).

The protein–protein interaction (PPI) network for SIL1 (STRING, Homo sapiens, confidence ≥0.70) recapitulated curated links to HSPA5 and ER proteostasis partners, positioning HSPA5 among the highest-confidence neighbors (Figure 4). Network topology (degree and betweenness) underscored the centrality of the SIL1–HSPA5 axis within ER quality-control pathways, supporting the plausibility that compromised SIL1 function could perturb BiP-mediated proteostasis [13]. Docking analysis revealed notable structural and interactional differences between the wild-type and mutant forms of the HSP5A protein. In the wild-type complex (Figure 5a), stable hydrogen bond networks were observed at the interaction interface, indicating a strong and specific binding affinity. However, in the mutant model (Figure 5b), several key hydrogen bonds were disrupted or rearranged, leading to an altered interaction pattern and reduced binding stability. These findings suggest that the variant in HSP5A may significantly affect its chaperone binding dynamics and functional conformation (Figure 5). These findings are consistent with previously reported in silico structural predictions and are interpreted as hypothesis-generating, along with the proposed insertion effect for *SIL1* gene c.453+1G>T [14,15].

A literature review concerning the clinical correlation of the study has been summarized. In the review, as in other literature publications, the clinical findings caused by homozygous variants leading to loss of function in the *SIL1* gene are consistent with our case presented in Table 2. The reported rates of frameshift-nonsense and splice variants in the literature are quite high.

## 4. Discussion

MSS is a rare autosomal recessive disorder marked by striking variability in how it presents from one patient to another. While the core triad of cerebellar ataxia, hypotonia, and congenital cataracts defines the disease, many patients also develop progressive weakness, skeletal deformities, and occasionally endocrine or cognitive involvement. The underlying cause traces back to pathogenic variants in *SIL1*, a gene encoding a nucleotide-exchange factor that works with HSPA5 (also known as BiP) to regulate protein folding within the ER. When SIL1 is disrupted, this delicate system fails, leading to an accumulation of misfolded proteins, activation of the UPR, and eventual cellular stress and degeneration—particularly in neurons and muscle tissue, where the demand for protein folding is exceptionally high. Although fewer than two hundred cases have been molecularly confirmed, each newly identified variant contributes to a more complete understanding of how this molecular defect translates into clinical diversity.

The patient described here carries a novel homozygous splice-site mutation, c.453+1G>T, located in intron 5 of *SIL1*. This variant has not been previously documented in ClinVar, LOVD, or any published series. The clinical picture in this patient—ataxia, hypotonia, and bilateral cataracts—matches the classic MSS profile; additional findings such as kyphoscoliosis, pectus excavatum, synophrys, and hypergonadotropic hypogonadism further support this diagnosis. These findings are not random additions; rather, they enrich the clinical vocabulary of *SIL1*-related disease, pointing to broader systemic involvement that has probably been under-recognized due to the rarity of such cases. It is through such detailed case-level observations that subtle but meaningful phenotypic nuances begin to emerge.

Most *SIL1* variants reported in the literature are insertion site or frameshift mutations, typically in exons 4, 6, 9, and 10, which splice the protein and eliminate its function [1,4,15,16,17,18,19,24]. The variant we describe expands this spectrum by introducing a pathogenic change within intron 5, upstream of the previously affected regions. Its predicted effect—loss of proper splicing—parallels other known splice-site mutations such as c.1029+1G>A and c.645+1G>A (Senderek et al. [4]), c.645+2T>C (Horvers et al. [8]), and c.453+5G>A (Rochdi et al. [22]). All of these ultimately compromise SIL1 stability, resulting in truncated proteins or transcript decay. This pattern supports a consistent pathomechanism across diverse genomic sites: the critical disruption of SIL1-HSPA5 interaction and subsequent ER stress that defines MSS [4,8,21,22].

Our patient’s background also provides an important population-level insight. While the c.506_509dupAAGA and c.936dupG variants have been linked to founder effects in Finnish and Swedish families [2,3], the current variant appears to have arisen independently in a non-Finnish population, underlining the global mutational heterogeneity of *SIL1*. Each newly discovered private mutation adds depth to our understanding of the disease’s molecular landscape and reinforces the need for genetic screening beyond known founder mutations, particularly in regions where MSS is likely underdiagnosed.

From a clinical standpoint, the case aligns closely with the features described in earlier reports. Cerebellar ataxia, hypotonia, and cataracts are universal hallmarks documented across studies by Senderek et al. [4], Anttonen et al. [2], Krieger et al. [3], Horvers et al. [8], Gatz et al. [19], Anttonen et al. [20], Rochdi et al. [22], and Faheem et al. [24]. Neuroimaging revealed cerebellar atrophy, consistent with the MRI patterns noted in Horvers et al. [8] and Krieger et al. [3]. The skeletal deformities in our patient echo the abnormalities reported by Takahata et al. [19] and Krieger et al. [3], but features such as kyphoscoliosis and pectus excavatum remain rare in the MSS literature. Similarly, synophrys has not previously been highlighted as part of the phenotype and may represent either an underreported morphological sign or the influence of additional modifying genetic factors.

Endocrine manifestations, though uncommon, are gaining recognition. Hypergonadotropic hypogonadism, seen in this patient, has been described sporadically in the literature, including in the reports by Karim et al. [17] and Krieger et al. [3]. Its reappearance across genetically confirmed cases suggests a more integral link between SIL1 dysfunction and endocrine pathways than previously assumed. Interestingly, while several patients in earlier studies exhibited cognitive delay or microcephaly [3,20,21,22], our patient’s normal cognitive function and developmental milestones indicate a milder neurological phenotype, suggesting that variant position and splicing severity may shape tissue-specific vulnerability. This again highlights the spectrum nature of MSS—one that ranges from relatively mild motor presentations to severe multisystemic disease.

Mechanistically, the c.453+1G>T variant likely disrupts SIL1’s ability to exchange ADP for ATP on HSPA5, halting the chaperone cycle and precipitating ER stress [16]. Because the affected site is positioned just upstream of exons encoding the ARM domains critical for BiP binding [6], it may influence the tertiary conformation of the downstream protein even if translation proceeds partially. HSPA5 acts as an ER chaperone and plays a key role in protein folding and quality control within the ER lumen. It assists the folding of secreted or membrane proteins and participates in the retention and triage of misfolded proteins [16,17]. In silico docking studies from this work suggested reduced binding affinity between mutant SIL1 and HSPA5, a finding consistent with the molecular alterations described by Hathazi et al. [21] and Rochdi et al. [22]. This defective interaction could impair neuronal migration and muscle fiber maintenance, explaining the ataxia and myopathy central to MSS pathophysiology [15].

When placed within the broader context of known MSS variants, this case falls into an intermediate severity range—broader in scope than the triad-only cases described by Senderek et al. [4] and Gatz et al. [19], yet milder than the multisystemic presentations reported by Krieger et al. [3] and Anttonen et al. [20]. The patient’s preserved intellect but notable skeletal and endocrine features reflect a delicate balance between genetic disruption and compensatory cellular mechanisms. Such variability underscores why no two MSS patients present identically, even when harboring similar classes of mutations.

From a diagnostic perspective, this case reinforces the importance of scrutinizing intron–exon boundaries during genetic evaluation. Splice-site defects like c.453+1G>T may evade standard exome pipelines, emphasizing the need for expanded sequencing and RNA-based confirmation. Functional studies—including RNA analysis, minigene assays, and SIL1-HSPA5 co-expression experiments—are crucial next steps to confirm transcript disruption and to define how these changes translate into cellular dysfunction. Beyond their diagnostic role, such studies can guide future therapeutic efforts aimed at stabilizing ER proteostasis, either through chemical chaperones or gene correction approaches.

In summary, the *SIL1* c.453+1G>T variant represents a novel intron 5 splice-site mutation that preserves the classical molecular signature of SIL1-related disease but adds new clinical detail to the expanding MSS narrative. Its discovery reinforces the central role of SIL1-HSPA5 dysfunction in disease pathogenesis and broadens the recognized range of clinical features to include subtle skeletal and endocrine involvement. As more variants like this are identified and functionally characterized, the story of MSS becomes not just one of genetic defect but of cellular adaptation, resilience, and diversity—a reminder that even within the confines of a single gene, the human body finds countless ways to tell its story.

### 4.1. Limitations

This study reports a single homozygous MSS case with a *SIL1* c.453+1G>T splice-donor variant. Our conclusions rely on in silico prediction and clinical–segregation evidence without RNA-level confirmation of the splicing outcome or functional assays of SIL1–HSPA5 coupling. Longitudinal phenotyping and external replication are not yet available. Novelty statements are contingent on database snapshots and should be interpreted with the stated access context. Collectively, these factors warrant caution and motivate experimental validation. All inferences are based on hypothetical models requiring RNA-level experimental validation.

### 4.2. Future Directions

Priorities include: (i) patient RNA (RT-PCR/Sanger or minigene) to map exon usage and confirm the predicted frameshift; (ii) ER-stress/UPR profiling in patient-derived cells (e.g., BiP, CHOP, XBP1 splicing); (iii) co-immunoprecipitation and nucleotide-exchange readouts to quantify SIL1’s NEF activity on HSPA5; (iv) rescue experiments with wild-type SIL1 in patient cells; (v) systematic genotype–phenotype aggregation to refine penetrance and spectrum in upstream-truncating *SIL1* variants (vi) demonstrating splice products in patient-derived cells through protein studies (Western blot, ELISA, etc.) will be critical to definitively distinguish between the two scenarios (cryptic donor utilization or splicing defect).

## 5. Conclusions

We present a novel homozygous c.453+1G>T splice-donor variant in SIL1 associated with a canonical MSS phenotype plus additional skeletal and endocrine features. The clinical picture, segregation, and ACMG evidence (PVS1, PM2, PP3) support a pathogenic loss-of-function classification, while the STRING network (Figure 4) provides a coherent, testable framework linking upstream truncation to impaired SIL1–HSPA5 coupling and ER proteostasis failure that future in vitro work should substantiate.

## Figures and Tables

**Table 1:** Pathogenicity of detected variant in the patient (↑: higher than normal value, ↓: lower than normal value).

Gene	*SIL1*
**Transcript (RefSeq)**	NM_022464.5
**Variant**	c.453+1G>T
**Dbsnp**	Novel
**Genomic coordinate**	chr5:138,378,308 (GRCh37/hg19)
**Exon/Intron**	Intron 5 (canonical + 1 splice-donor)
**Variant type**	Splice donor variant
**Zygosity**	Homozygous
**Inheritance/Segregation**	Autosomal recessive; parents heterozygous carriers (Aa); sibling AA unaffected (reported)
**dbSNP**	Not listed
**ClinVar**	Not found
**LOVD**	Not found
**gnomAD (exomes/genomes)**	Not found
**Protein domain**	Nucleotide exchange factor (Fes1-like)
**Conservation**	Conserved
**Computational—dbscSNV**	Pathogenic (score not provided)
**Computational—MaxEntScan**	Pathogenic (Δscore not provided)
**Computational—BayesDel**	Pathogenic
**Computational—DANN**	Pathogenic
**Computational—SpliceAI**	Not reported
**Computational—CADD**	Not reported
**Aggregated prediction**	Pathogenic
**ACMG evidence**	PVS1, PM2, PP3
**ACMG classification**	Pathogenic
**Phenotype summary**	Hypergonadotropic hypogonadism: FSH 50.53 mIU/mL (↑), LH 40.64 mIU/mL (↑), total testosterone 209.9 ng/dL (↓); thyroid axes within reference.

**Table 2:** Reported variants and clinics of the patient in the literature.

Reference	SIL1 Variant (NM_022464.5)	Zygosity	Exon(s)	Eye Findings	Brain Abnormality	Skeletal Findings	Development	Muscle Findings	Other Findings
Senderek et al., 2005 [4]	c.1029+1G>A; c.645+1G>A	HM	6, 9	congenital cataracts	cerebellar atrophy	short stature	psychomotor delay	myopathic EMG; ataxia; hypotonia	
Anttonen et al., 2005 [2]	c.331C>T; c.212dupA; c.506_509dupAAGA; (c.506_509dupAAGA+645+2T>C CHT)	HM	4, 9	bilateral cataracts	cerebellar atrophy	short stature		unstable gait; ataxia; loss of ambulation (~20 yrs)	
Karim et al., 2006 [17]	c.1312C>T (p.Gln438Ter)	HM	10	cataracts ±			delayed motor & mental development	progressive weakness; hypotonia, cerebellar ataxia	hypogonadism
Takahata et al., 2010 [18]	c.603_607del (p.Glu201AspfsTer6)	HM	6	cataracts;			spasticity	Ataxia; skeletal deformities; myopathy; hypotonia	
Krieger et al., 2013 [3]	Multiple (19 variants incl. c.16C>T; c.936dupG; c.1312C>T; c.1370T>C)	HM/CHT	4, 6, 9, 10	congenital cataracts	microcephaly	skeletal deformities	psychomotor delay	hypotonia; ataxia; ↑ CK	hypogonadism
Horvers et al., 2013 [8]	c.1060C>T (p.Gln354Ter); c.935G>A (p.Gly312Glu); c.645+2T>C	HM/CHT	6, 9, 10	cataracts (3–5 yrs)	cerebellar atrophy	skeletal deformities	mild intellectual disability	ataxia; myopathy	
Gatz et al., 2019 [19]	c.1370T>C (p.Leu457Pro)	HM	10	dysarthria; congenital cataracts	microcephaly		psychomotor delay	limb/truncal ataxia; hypotonia	
Anttonen et al., 2008 [20]	c.1042dupG (p.Glu348GlyfsTer4)	HM	9	hyperopia vs. cataracts; strabismus; nystagmus			global developmental delay	cerebellar ataxia; myopathy	
Hathazi et al., 2021 [21]	c.645+1G>A; c.947_948insT; c.1030–18G>A	HM/CHT	6, 10	cataracts	microcephaly; cerebellar atrophy		intellectual disability	progressive myopathy	overlap with INPP5K phenotype
Rochdi et al., 2022 [22]	c.453+5G>A	HM	6	bilateral cataracts		mild skeletal abnormalities	intellectual disability	cerebellar ataxia; hypotonia	
Faheem et al., 2024 [23]	c.936dupG (p.Leu313AlaFsTer39)	HM	9	cataracts; strabismus	cerebellar atrophy			ataxia; hypotonia; abnormal gait; ↑ CK	
Our study	c.453+1G>T (novel)	HM	5	bilateral cataracts		kyphoscoliosis; pectus excavatum	mild intellectual disability	cerebellar ataxia; hypotonia	hypergonadotropic hypogonadism; synophrys

HM: Homozygous Mutation; CHT: Compound Heterozygous Mutation; ±: Feature Variably Present, ↑: higher then normal value.

## Data Availability

De-identified sequencing analyses and bioinformatic outputs (e.g., variant call/annotation reports, STRING network files, and structural modeling outputs) that support the findings of this study are available from the corresponding author upon reasonable request and subject to ethical approval.

## Abbreviations

ACMG	American College of Medical Genetics and Genomics
AA	Amino Acid
ARM	Armadillo Repeat Motif
ATP	Adenosine Triphosphate
BIP	Binding Immunoglobulin Protein
B@MER	Biruni Research Center
CADD	Combined Annotation Dependent Depletion
CHOP	C/EBP Homologous Protein
C-SCORE	Confidence Score
DBSCSNV	Database for Splicing Consensus SNVs
DNA	Deoxyribonucleic Acid
ER	Endoplasmic Reticulum
FLAIR	Fluid-Attenuated Inversion Recovery
FSH	Follicle-Stimulating Hormone
GRP78	Glucose-Regulated Protein 78
HGNC	HUGO Gene Nomenclature Committee
HSPA5	Heat Shock Protein Family A (Hsp70) Member 5
I-TASSER	Iterative Threading Assembly Refinement
LP	Likely Pathogenic
LH	Luteinizing Hormone
LOVD	Leiden Open Variation Database
MAF	Minor Allele Frequency
MRI	Magnetic Resonance Imaging
MSS	Marinesco–Sjögren Syndrome
NCBI	National Center for Biotechnology Information
NEF	Nucleotide Exchange Factor
NMR	Nuclear Magnetic Resonance
OMIM	Online Mendelian Inheritance in Man
PPI	Protein–Protein Interaction
PP3	Supporting Pathogenicity Criterion 3
PVS1	Pathogenic Very Strong 1
RNA	Ribonucleic Acid
RT-PCR	Reverse Transcription Polymerase Chain Reaction
SIL1	SIL1 Nucleotide Exchange Factor
STRING	Search Tool for the Retrieval of Interacting Genes/Proteins
UPR	Unfolded Protein Response
VUS	Variant of Uncertain Significance
WES	Whole-Exome Sequencing
WT	Wild Type
XBP1	X-Box Binding Protein 1
